# Case Report: Cord blood-derived natural killer cells as new potential immunotherapy drug for solid tumor: a case study for endometrial cancer

**DOI:** 10.3389/fimmu.2023.1213161

**Published:** 2023-06-30

**Authors:** Yongxu Mu, Jiabei Tong, Yujun Wang, Yuxiao Yang, Xiaoyun Wu

**Affiliations:** ^1^ Department of Interventional, The First Affiliated Hospital of Baotou Medical College, Inner Mongolia University of Science and Technology, Baotou, China; ^2^ Department of Biomedical Sciences, Faculty of Medicine and Health Sciences, University Putra Malaysia, Serdang, Selangor, Malaysia; ^3^ Department of Technology, Beijing Stem Cell(ProterCell) Biotechnology Co., Ltd., Beijing, China; ^4^ Department of Technology, Inner Mongolia Stem Cell(ProterCell) Biotechnology Co., Ltd., Hohhot, China; ^5^ Department of Technology, Research Center for Hua-Da Precision Medicine of Inner Mongolia Autonomous Region, Hohhot, China

**Keywords:** natural killer, endometrial cancer, advanced malignancies, cord blood (CB) donor, immunotherapy

## Abstract

Adoptive transfer of natural killer (NK) cells represents a viable treatment method for patients with advanced malignancies. Our team previously developed a simple, safe, and cost-effective method for obtaining high yields of pure and functional NK cells from cord blood (CB) without the need for cell sorting, feeder cells, or multiple cytokines. We present the case of a 52-year-old female patient diagnosed with poorly differentiated stage IVB (T3N2M1) endometrial cancer, who exhibited leukemoid reaction and pretreatment thrombocytosis as paraneoplastic syndromes. The patient received two courses of CB-derived NK (CB-NK) cell immunotherapy between March and September 2022, due to her extremely low NK cell activity. Two available CB units matched at 8/10 HLA with KIR-mismatch were chosen, and we were able to produce NK cells with high yield (>1.0×10^10^ NK cells), purity (>90%), and function (>80%) from CB without cell sorting, feeder cells, or multiple cytokines. These cells were then adoptively transferred to the patient. No adverse effects or graft-versus-host disease were observed after infusion of CB-NK cells. Our clinical experience supports the efficacy of CB-NK cell treatment in increasing NK cell activity, depleting tumor activity, improving quality of life, and reducing the size of abdominal and pelvic masses with the disappearance of multiple lymph node metastases through the regulation of systemic antitumor immunity. Remarkably, the white blood cell and platelet counts decreased to normal levels after CB-NK cell immunotherapy. This clinical work suggests that CB-NK cell immunotherapy holds promise as a therapeutic approach for endometrial cancer.

## Introduction

Endometrial cancer (EC) is a major global health concern, with increasing incidence and disease-related mortality rates. Despite standard treatments for advanced or recurrent EC, the efficacy and prognosis remain poor, highlighting the need for innovative therapies. Natural killer (NK) cells, which possess innate tumor-killing abilities, have shown promise as a potential cancer therapy. Dysfunctions in NK cells, such as decreased cell numbers and impaired cytotoxicity, have been observed in EC patients, motivating researchers to investigate adoptive NK cell-based immunotherapy protocols to enhance NK cell anti-tumor responses (1–3).

Early investigations using autologous NK cells for cancer therapy did not yield satisfactory clinical outcomes (4). Allogeneic NK cell therapy, which utilizes donor-recipient incompatibility in KIR and HLA, has been shown to enhance the anti-cancer efficacy of NK cells (5). Cord blood (CB) is a readily available source of allogeneic NK cells for therapeutic infusion. The existence of CB Banks provides a potential platform for selecting donors with specific HLA haplotypes to generate alloreactive NK cells, which may have improved effectiveness in targeting host tumor cells (6, 7). This makes CB-derived NK cells an attractive option for third-party therapy, given the large availability of stored and fresh CB units.

Natural killer (NK) cells can be derived from CD56^+^ NK cells or CD34^+^ hematopoietic stem and progenitor cells (HSPCs) in CB. Our previous research yielded a novel method for obtaining high yield, purity, and functionality NK cells from CB without the use of cell sorting and feeder cells/multiple cytokines (PCT/CN2016/081198) (8, 9). This approach is cost-effective, easy to standardize, and has practical advantages for the development of cellular drugs. However, the culture medium in this method contains components of animal or human serum, which necessitates changes to adapt it for clinical use. As a result, we have developed a clinical-grade chemically defined serum-free and xeno-free medium (Patent pending, culture medium components and mechanism will be discussed in detail in our preparing article) as a substitute for animal or human serum. This serum-free medium is suitable for *in vitro* preparation of NK cells and meets the standards required for a potential therapeutic product, making it more accessible for clinical practice and increasing safety (10). Preclinical studies have demonstrated that CB-NK cells can be produced in sufficient numbers with effective tumor cytotoxicity, leading to the translation of several preclinical CB-NK cell studies into good manufacturing practice quality clinical-scale manufacturing of NK cells that are used in clinical trials. In this study, we utilized CB-NK cells as a novel form of immune cell therapy for the treatment of EC, marking the first application of CB-NK cells for this purpose.

## Case presentation

A 52-year-old female patient presented with a history of irregular vaginal bleeding for 2 years and abdominal pain for 1 month. Imaging with chest-abdomen-pelvis Computed Tomography (CT) scan revealed a space-occupying lesion measuring 175 × 138 × 136mm in the abdomen and pelvis, raising suspicion for malignancy. Multiple enlarged lymph nodes were identified in the mediastinum, bilateral supraclavicular area, retroperitoneal area, and pelvic cavity, with the possibility of metastasis to the lungs, left psoas major muscle, and bilateral upper ureter. Positron Emission Tomography-CT (PET-CT) confirmed the presence of these lesions and also revealed pleural lesions associated with costal erosive metastases. Further evaluation with histopathology and immunohistochemical staining of a biopsy confirmed the diagnosis of poorly differentiated EC stage IVB (T3N2M1), with tumor markers showing elevated human epididymis protein 4 (HE4, 461.4pmol/L) and carbohydrate antigen 125 (CA125, 181.1U/mL) levels. Routine blood tests indicated leukemoid reaction (LR) with elevated white blood cell (WBC, 18.94 × 10^9^/L) and platelet (PLT, 984 × 10^9^/L) counts and low hemoglobin level (Hb, 63g/L), as well as pretreatment thrombocytosis confirmed by bone marrow aspirates and biopsy. There was no known family history of colon cancer, and endometrial cancer related to Lynch syndrome was ruled out.

**Figure 1:**
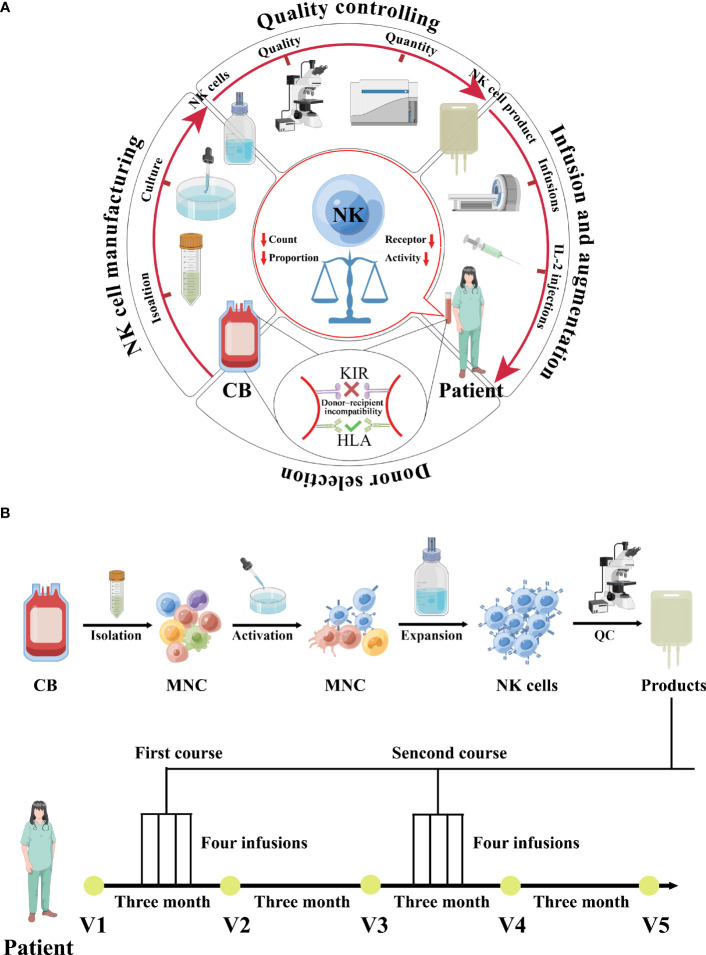
A schematic diagram depicting the clinical-grade manufacturing process of NK cell products from CB, as well as the schedules for CB-derived NK cell treatments and immunophenotype monitoring, is shown. **(A)** A schematic diagram depicting the process of CB-derived NK cell-based immunotherapy, which includes donor selection, NK cell manufacturing, quality control, adoptive transfer of NK cells to the patient, and *in vivo* NK cell augmentation. **(B)** A schematic diagram depicting the clinical-grade manufacturing process of NK cell products from CB, as well as the schedules for CB-derived NK cell treatments and immunophenotype monitoring, is shown. The patient was enrolled in February 2022 and received cell treatments beginning in March 2022. Two treatment courses were administered, each consisting of four infusions given over four days, with one course in March and the other in September. Immunophenotyping of the patient was performed before and after each round of NK cell treatments.

Complete surgical excision was not feasible for the 52-year-old female patient with poorly differentiated EC stage IVB (T3N2M1) as the tumor had metastasized to other organs. Chemotherapy was discontinued due to intolerable adverse effects. Further evaluation showed that the patient had reduced lymphocyte proportion (6.73%) and count (1413.3cells/μL) with extremely low NK cell activity (78pg/mL), which was confirmed via the test-kit manufacturer given reference ranges (defined <100 as extremely low, 100–250 as very low, 250–500 as low, and ≥500 as normal). As a result, the patient was enrolled in a clinical trial comprising CB-NK cells for EC. CB-NK immunotherapy requires multiple steps, including donor selection, NK cell manufacturing, quality control, adoptive transfer of NK cells to the patient, and NK augmentation *in vivo* to achieve therapeutic effects due to the scarcity of NK cells in peripheral circulation (**Figure 1A**).

### Donor selection HLA and KIR genotyping

Donor selection for CB-NK immunotherapy is critical and is based on KIR and HLA typing because their polymorphisms affect NK cell function and clinical outcomes (11). HLA genotyping of patients and CB donor was performed using LinkSeq HLA typing kits (Linkage Biosciences, San Francisco, CA, USA) and HLA-FluoGene kits (inno-train Diagnostik, Kronberg, Germany). KIR genotyping of CB donors and patients was performed using the LinkSeq KIR typing kit (Linkage Biosciences). Two available CB units matched at 8/10 HLA loci (HLA-A, -B, -C, -DR, -DQ) with KIR mismatch were chosen for this patient (**Supplementary Data Tables 1, 2**).

### NK cell manufacturing

The NK cells were activated and expanded under good manufacturing practice (GMP) conditions (**Figure 1A**). Briefly, mononuclear cells were isolated from CB using Ficoll-Hypaque density gradient centrifugation. For activation, mononuclear cells were cultured for 3 days in T175 flasks at 2 × 10^6^ cells/mL in serum free media supplemented with 2000 IU IL-2/mL (Four Rings Biopharma, China), 0.01KE/mL group A streptococcus (Lu Ya Pharma, China) and 5 μM zoledronate (Novartis Pharma). For expansion, the fresh medium containing 2000 IU IL-2/mL was added every 2 to 3 days. At the end of culture (19-23 days), cells were harvested, washed twice with 0.9% saline solution/2% human serum albumin (HSA; ZLB Behring AG, Bern, Switzerland) and diluted in 100 mL saline solution 0.9%/2% HSA.

### Quality control and release criteria

The quality and quantity of the NK cell product were evaluated, including expansion, purity, potency, and safety (**Figure 1A**). Quality controls were regularly performed with samples taken on day 0, day 7, day 14, and days 19-23 of culture. Sterility was tested with the BacT/Alert FN/FA microbial detection system (Biomerieux, Inc, Durham, NC, USA). Viability was determined at several time points during expansion culture using the Live/Dead fixable aqua dead cell stain kit (Invitrogen Molecular Probes, Eugene, OR, USA). Cellular composition of cultures was assessed by flow cytometric single platform analysis. Cells were stained with anti-CD3 and CD56 antibodies. NK cells were analyzed by flow cytometry for the expression of inhibitory receptors NKG2A, PD-1, TIM-3, TIGIT, or LAG-3 (BioLegend). Samples taken from four of all expansion cell bags were used to estimate the total number and expansion fold of NK cells in the entire expansion culture.

For functional assay, NK cells were co-cultured with EC target cell line—Ishikawa labeled with 1 mM CFSE for a total of 5 h at E:T ratio of 10:1, 5:1 and 2:1. Following incubation, cells were stained with 7-AAD (Becton–Dickinson) for 10 min, washed, and fixed with 1% PFA. Flow cytometric analysis of cells was performed within 1 h. The CD107a expression was performed for NK degranulation using a CD107a detection kit (MBL, Nagoya, Japan) according to the manufacturer’s instructions (12).

The release criteria of the NK cell product were defined as, minimally, the NK cell purity, sterile, undetectable Mycoplasma (Lonza, Rockland, ME) and endotoxin level of <2 EU/mL (Cambrex, Walkersville, MD) and a viability of >90%.

Using this clinically applicable methodology, overall fold expansions within approximately 3 weeks were 47.3 and 37.1 from donors 1 and 2, respectively. Because the initial percentages of CD56^+^CD3^-^ NK cells were 11.7 and 13.2 from donors 1 and 2, respectively, and the final percentages of NK cells in the expanded populations were 93.7 and 91.6, the overall NK cell expansions were 378.8 and 257.5, respectively (**Supplementary Data Table 1** and **Figure 1A**). We have previously observed a significant upregulation in the expression of activating receptors during culture (8, 9), whereas most inhibitory receptors remained unchanged, except for PD-1 and LAG-3 that were slightly elevated (**Supplementary Data Figure 1B**). CB-NK cells had higher CD107a expression, suggesting more degranulation (**Supplementary Data Figure 1C**). Moreover, CB-NK cells efficiently lysed EC cell line-Ishikawan reaching 82.4% and 86.1% at effector/target ratio 10:1, respectively (**Supplementary Data Table 1** and **Figure 1D**).

### CB-NK treatment

100 ml of the NK cell product was infused into the patient within 1-hour post-collection by intravenous administration over 1 hour in an outpatient setting, followed by continuous intravenous infusion of recombinant human interleukin-2 (2 × 10^6^ IU/m^2^/day) for 4 days. The therapeutic schedule and regimen for CB-NK cell immunotherapy are illustrated in **Figure 1B**. The patient received two courses of treatment, with the first course administered in March 2022 and the second in September 2022. Each course consisted of intravenous transfusion once daily for four consecutive days (**Figure 1B** and **Supplementary Data Table 1**).

## Clinical results

### Response to CB-NK cell therapy

**Figure 2:**
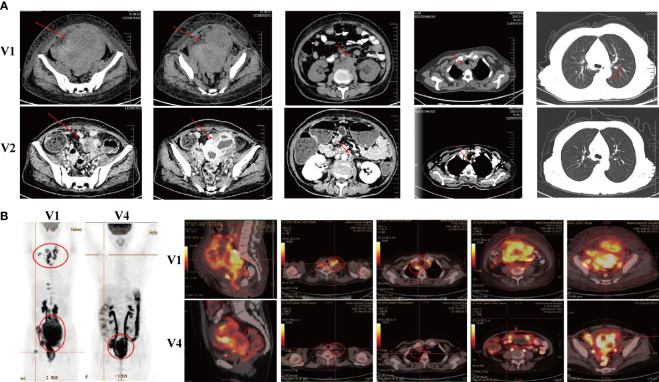
Radiological evaluations of the patient at different time points. **(A)** CT scans were conducted before (V1) and approximately two months (V2) after the initial treatment course. **(B)** PET-CT scans were performed before (V1) and approximately eight months (V4) after the initial treatment course.

**Figure 3:**
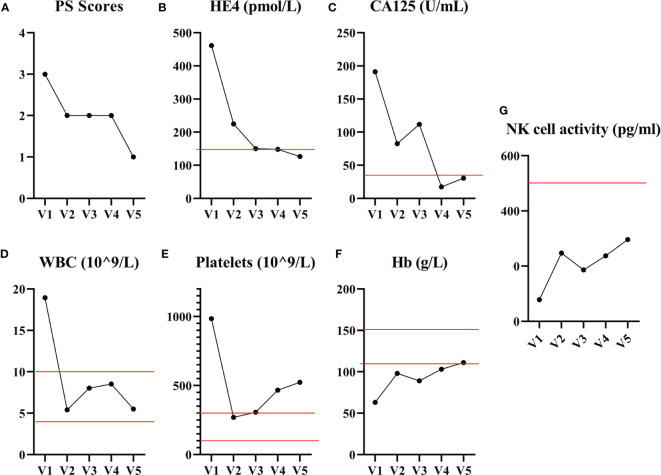
The performance status score and blood biochemical examination results before and after CB-NK cell treatments are presented. **(A)** The performance status score of the patient decreased from 3 to 1 after receiving two courses of CB-NK cells treatment. The tumor markers HE4 **(B)** and CA125 **(C)** levels declined rapidly and remained within the normal range throughout the treatment. The WBC **(D)** and PLT **(E)** counts decreased to the reference range, while Hb **(F)** levels increased to the reference range with the use of CB-NK cell-based immunotherapy. Additionally, NK cell activity **(G)** increased gradually throughout the two-course treatment. Red line represents the reference value.

Following the first cycle of CB-NK cells immunotherapy, the patient achieved a partial response. Improvements were noted in her physical strength, appetite, and sleep quality. A CT scan was performed to assess the efficacy of CB-NK cells immunotherapy, revealing a significant reduction in abdominal and pelvic masses, as well as in multiple enlarged lymph nodes in various regions. However, some lymph nodes around the right iliac vessels and bilateral axillary lymph nodes had slightly increased in size, and multiple nodules in both lungs were still present, indicating possible metastasis (**Figure 2A**). To further evaluate the therapeutic effect on the progressive tumor, a PET-CT scan was performed after the second cycle of CB-NK cells immunotherapy. The scan showed a reduction in the size of the hypermetabolic mass at the right side of the uterus, and the metabolism and volume of the uterus were significantly reduced compared with pre-treatment PET-CT imaging. Although the mass at the right femoral head and the bone metastases in the right 10th and 11th ribs remained unchanged, the metabolism of bone metastases returned to normal. Multiple lymph node metastases also disappeared in the neck, mediastinum, retroperitoneum, and pelvis (**Figure 2B**). The patient’s quality of life improved significantly, as indicated by a decrease in the performance status score from 3 to 1 after two courses of CB-NK cells treatment (**Figure 3A**). Tumor markers HE4 and CA125 (**Figures 3B, C**), as well as blood parameter WBC and PLT (**Figures 3D, E**), declined rapidly, while Hb increased to normal levels with the use of CB-NK cell-based immunotherapy (**Figure 3F**). Importantly, NK cell activity increased gradually through the two-course treatment (**Figure 3G**). No side effects, such as fever, vomiting, or convulsions, were observed during or after the injection of CB-NK cells. Furthermore, no signs of graft-versus-host disease (GVHD) were reported during the entire follow-up period.

### Immunophenotypic changes

**Figure 4:**
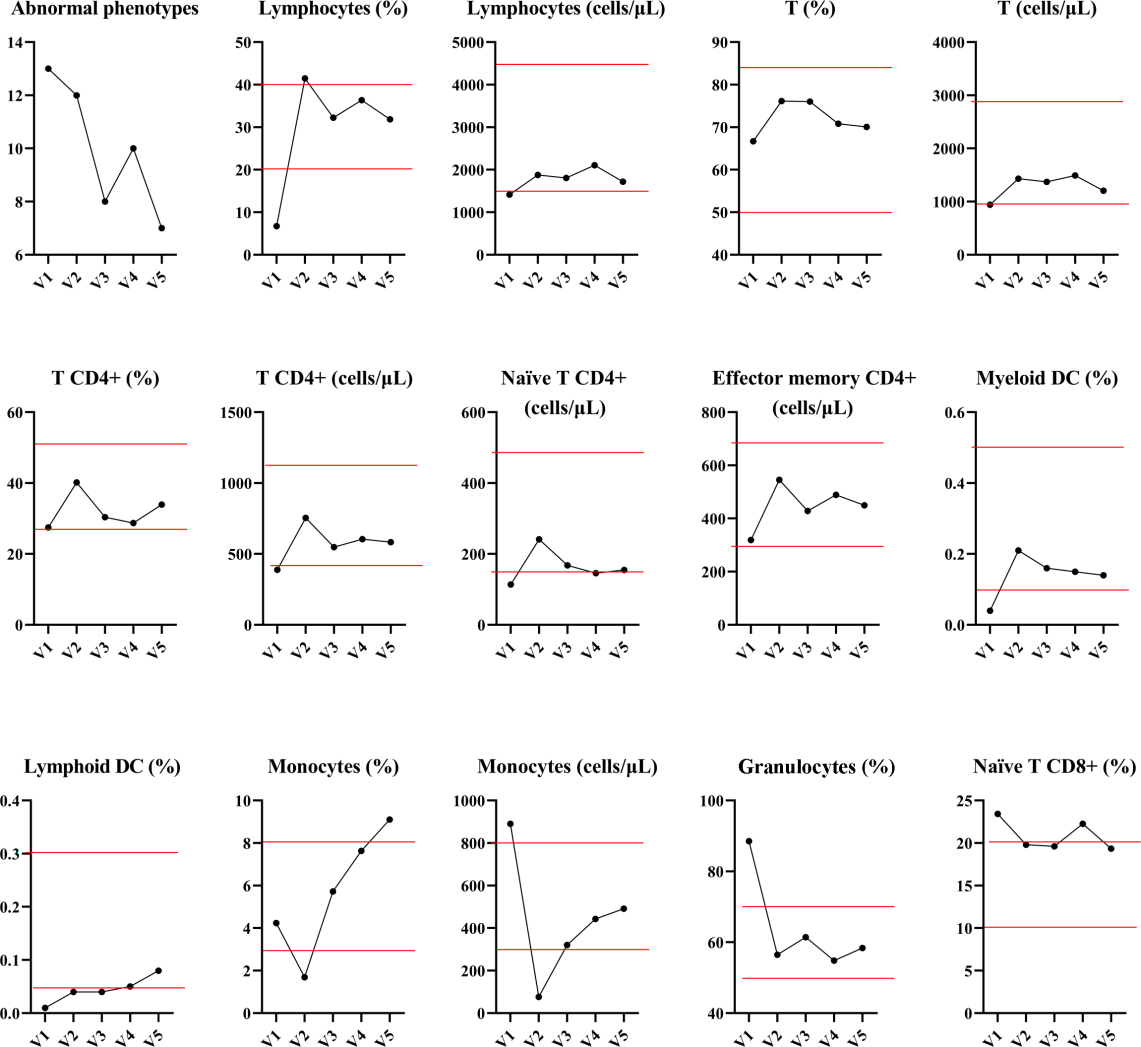
The changes in immunophenotyping before and after CB-NK cell treatments were analyzed. The results revealed a marked improvement in immunity, evidenced by a decrease in the number of abnormal phenotypes. Administration of CB-NK cells was associated with a significant increase in lymphocytes (CD14^Low^), T cells (CD3^+^), helper T cells (CD3^+^CD4^+^), naïve CD4^+^T cells (CD3^+^CD4^+^CD27^+^CD45RA^-^), effector memory CD4^+^T cells (CD3^+^CD4^+^CD27^-^CD45RO^+^), myeloid dendritic cells (DC, Lineage^-^CD11c^+^HLA-DR^+^), and lymphoid DC (Lineage-CD123^+^ HLA-DR^+^). Furthermore, CB-NK cell therapy was also associated with a decrease in the number of monocytes (CD14^+^) and granulocytes (CD14^high^) and naïve CD8^+^T cells. Red line represents the reference value.

In order to elucidate the mechanisms underlying the immune response mediated by CB-NK cells, we conducted an analysis of immunophenotypic changes using flow cytometry. The results of this study indicate that CB-NK cell therapy significantly enhances the immune system by reducing the abnormal phenotypes associated with disease. Specifically, administration of CB-NK cells was associated with an increase in the number of lymphocytes (CD14^Low^), T cells (CD3^+^), helper T cells (CD3^+^CD4^+^), naïve CD4^+^T cells (CD3^+^CD4^+^CD27^+^CD45RA^-^), effector memory CD4^+^T cells (CD3^+^CD4^+^CD27^-^CD45RO^+^), myeloid dendritic cells (DC, Lineage^-^CD11c^+^ HLA-DR^+^), and lymphoid DC (Lineage^-^CD123^+^HLA-DR^+^), as well as a decrease in the number of monocytes (CD14^+^), granulocytes (CD14^high^), and naïve CD8^+^T cells (CD3^+^CD4^+^CD27^+^CD45RA^-^, **Figure 4** and **Supplementary Data Table 3**).

The results of our study indicate that CB-NK cell-based immunotherapy may have a beneficial effect in the treatment of EC by modulating T cell responses mediated by DC, leading to the development of systemic antitumor immunity and subsequent reduction in tumor size.

## Discussion

To our knowledge, there have been only a few reported cases of EC with LR, which were mainly found in advanced stages and had a poor prognosis (13). Malignant tumors with LR typically respond poorly to chemotherapy, resulting in a short survival time for patients (14). Pretreatment thrombocytosis is associated with reduced overall survival and disease-free survival in many solid tumors, including EC. Thrombocytosis is also correlated with adverse clinicopathological parameters, which demonstrate that thrombocytosis predicts a poor prognosis in patients with EC (15). Paraneoplastic syndromes, such as LR and thrombocytosis, are rarely observed in gynecological tumors, particularly in EC. Treatment strategies for cancer with LR and thrombocytosis are limited and are rarely reported in the literature. Here, we report a rare case of an EC patient with LR and pretreatment thrombocytosis as paraneoplastic syndromes, who simultaneously received two courses of CB-NK cell immunotherapy (a total of 8 infusions). We clinically demonstrated that CB-NK cell treatments positively regulated the patient’s peripheral immune functions, increased NK cell activity, depleted tumor activity, improved quality of life, and remarkably reduced the size of abdominal and pelvic masses with the disappearance of multiple lymph node metastases. Surprisingly, in this case, the WBC and PLT counts decreased to normal levels after CB-NK cell immunotherapy. Our clinical work suggests that CB-NK cell immunotherapy could be developed into a promising therapy for EC. The investigational new drug for our CB-NK cell application in clinical therapy is currently undergoing approval in China.

Several methods have been developed for expanding NK cells in clinical grade enrichment protocols. These methods involve immunomagnetic selection of NK cells or immunomagnetic depletion of T cells and/or B cells, resulting in a pure cell therapy product (16). A clinical study using this method to pretreat adoptively transferred CB-NK cells into multiple myeloma patients showed a clinical response in 10 of 12 patients, among which 8 had complete remission (17). Based on this finding, a phase II study of this therapy is currently in progress (NCT01729091). Furthermore, the direct immunomagnetic selection of CD34^+^ HSPC is another approach for producing NK cell therapy products. Celularity Company recently announced Investigational New Drug approval for CB-NK cell product from the U.S. Food and Drug Administration to treat multiple myeloma, acute myeloid lymphoma, and glioblastoma multiforme (https://celularity.com/nk-cell-platform/ ). Currently, several clinical trials are ongoing to evaluate the efficacy of CB derived HSPC-NK cell therapies in hematological and solid tumors (NCT04489420, NCT04310592, NCT04309084). To optimize ex vivo NK cell expansion protocols, it is crucial to fine-tune the enrichment of NK cells or HSPC, use feeder cells or optimize the cytokine cocktail. Typically, a dose of CB or placenta donor can be expanded to an amount sufficient for one adoptive transfer procedure (1-3×10^9^ NK cells). We have developed a method to obtain high yield, purity, and functionality NK cells from CB mononuclear cells without cell sorting and feeder cells/multiple cytokines. Three weeks of culture of one CB unit yields a sufficient amount of >1.0×10^10^ NK cells with a purity of >90%, equivalent to four doses. Therefore, we performed four cell infusions from a CB unit in this study.

In allogeneic NK cell-based therapy, the presence of contaminated T cells raises concern due to the potential for adverse effects stemming from GVHD. One approach to address this issue is the depletion of CD3-expressing cells following expansion of NK cells, limiting contaminating total T cells to less than 1-5×10^5^/kg (10). Despite obtaining a high purity NK cell product using our method, the number of contaminated T cells exceeded the minimum standard. However, our analysis showed that the contaminated T cells were immature compared to mature T cells in peripheral blood. To avoid GVHD caused by alloreactive T cells, we chose two CB units with a matching of 8/10 HLA and immature contaminated T cells. We demonstrated the safety of administering >2×10^9^ NK cells per dose and up to 1.8×10^10^ in total, without any signs of GVHD or other local or systemic side effects. In allogeneic NK cell infusion therapy, mismatches between inhibitory KIR expressed on donor NK cells and recipients’ HLA ligands can trigger alloreactivity of NK cells (18). A study in the KIR-mismatched HSPC transplantation demonstrated alloreactivity NK cells against leukemia with no GVHD and no effects on engraftment. Of particular note, KIR-mismatched NK cells significantly augmented the five-year survival probability among patients with leukemia (19). Expanding this approach to the non-transplant setting resulted in a remission in 75% of poor-prognosis leukemia patients with a KIR-mismatch, whereas only 13% of those without such a mismatch (20). The choice of NK cell donor is still under investigation, and no specific setting has been clearly determined to be superior to others. For this patient, whose HLA typing included two HLA-C alleles belonging to different groups (C1 and C2 homozygous) and one HLA-B allele belonging to the Bw4 group, we attempted to select two CB units with KIR-B haplotype (>2 B activating gene loci). This choice is justified by the demonstrated enhancing clinical benefits of using HLA-C mismatch and KIR-B/X donors in other settings, particularly acute myeloid leukemia (21, 22).

Low NK cell activity is associated with a poor prognosis in patients with solid and non-solid tumors, whereas the presence of highly activated NK cells is correlated with improved prognosis (23). In patients with EC, the circulating activity level of NK cells was extremely low and gradually increased through two courses of CB-NK cell treatment, indicating that functionality was being restored (24, 25). The change in lymphocyte subsets suggested an improvement or decrease in immune function. Previous studies have proposed that NK cells can regulate other immune cells, including potentiating the functions of CD4^+^ and CD8^+^ T cells, DC, and activating neutrophils (26, 27). The elevation of CD4^+^ and CD8^+^ T cell differentiation and DC maturation observed in our study is consistent with previous findings, but the effect on granulocytes is inconsistent, which may be related to the source of NK cells or disease status. Interestingly, our work revealed that CB-NK cells were able to reduce the proportion and count of B cells, which is consistent with other findings (28). These results suggest a dynamic impact of CB-NK cell treatment on both innate and adaptive immune responses.

In conclusion, this case report describes the first use of CB-NK cell immunotherapy in EC treatment. The clinical outcome demonstrated that CB-NK cell therapy was safe and effective in treating EC. This promising trial opens a new avenue for cancer immunotherapy and may inspire further clinical studies using CB-NK cells. CB-NK cells have the potential to become a valuable “immune drug” for malignant tumors. This report represents a significant advancement in cancer research and treatment and may pave the way for future immunotherapeutic innovations in the field.

## Data availability statement

The raw data supporting the conclusions of this article will be made available by the authors, without undue reservation.

## Ethics statement

The studies involving human participants were reviewed and approved by Institutional Review Board of the First Affiliated Hospital of Baotou Medical College. The patients/participants provided their written informed consent to participate in this study. Written informed consent was obtained from the individual(s) for the publication of any potentially identifiable images or data included in this article.

## Author contributions

YM and YW designed and supervised the clinical study, enrolled the patient and took care of the patient. Moreover, they also supervised the CB-NK cell production for preclinical quality control. JT collected clinical data, performed the experiments, and performed statistical analyses. YY wrote and revised the manuscript and figure. YW edited and formatted the document. All authors contributed to the article and approved the submitted version.
