# Supplementary material for: Case Report: Cord blood-derived natural killer cells as new potential immunotherapy drug for solid tumor: a case study for endometrial cancer
Source: Front Immunol. 2023 Jun 30;14:1213161. doi: 10.3389/fimmu.2023.1213161 (PMC10348479; doi:10.3389/fimmu.2023.1213161)
Supplement: Supplementary file 1 [file Table_1.docx]

Supplementary Table 1 | CB-NK cells used in this study.

| NK Cell | Specification | Donor 1 | Donor 2 |
| --- | --- | --- | --- |
| Donor selection | | | |
| HLA-A, -B, -C, -DR, -DQ | ≥ 6/10 | 8/10 | 8/10 |
| KIR | mismatch | mismatch | mismatch |
| NK cell manufacturing | | | |
| Cell numbers (×10^8^ cells) | initial | 2.6 | 3.1 |
| % CD56^+^CD3^−^ cells | initial | 11.7 | 13.2 |
| Cell numbers (×10^8^ cells) | before infusion | 123 | 115 |
| % CD56^+^CD3^−^ cells | before infusion | 93.7 | 91.6 |
| Overall expansion fold | before infusion | 47.3 | 37.1 |
| NK cell expansion fold | before infusion | 378.8 | 257.5 |
| % CD3^+^ cells | before infusion | 2.4 | 3.8 |
| CD3^+^ cell numbers (×10^8^ cells) | before infusion | 2.9 | 4.4 |
| Quality controlling | | | |
| % CD56^+^CD3^−^ cells | > 80 | 93.7 | 91.6 |
| Endotoxin (EU/mL) | < 2 | < 0.25 | < 0.25 |
| Mycoplasma | negative | negative | negative |
| Sterility (bacterial) | negative | negative | negative |
| Cell viability | > 90% | 98.6% | 98.2% |
| Cell-mediated cytotoxicity | NK : Ishikawa=10 : 1 | 82.4% | 86.1% |
| Treatment Scheme | | | |
| Total infusion/injection times | N/A | 4 | 4 |
| Infusion frequency | N/A | once a day | once a day |
| Dose level (×10^9^ cells per infusion) | 4 > dose level > 2 | 2.6, 2.9, 3.3, 3.5 | 2.2, 3.1, 3.5, 2.7 |
