# Supplementary material for: Case Report: Cord blood-derived natural killer cells as new potential immunotherapy drug for solid tumor: a case study for endometrial cancer
Source: Front Immunol. 2023 Jun 30;14:1213161. doi: 10.3389/fimmu.2023.1213161 (PMC10348479; doi:10.3389/fimmu.2023.1213161)
Supplement: Supplementary file 2 [file Table_2.docx]

Supplementary Table 2 | KIR and HLA genotyping of patient and donors

| **Sample** | | | **HLA genotyping** | | | | | | | | | | | | | | **KIR genotyping** | | | | | | | | | | | | **KIR-ligand mismatch** |
| --- | --- | --- | --- | --- | --- | --- | --- | --- | --- | --- | --- | --- | --- | --- | --- | --- | --- | --- | --- | --- | --- | --- | --- | --- | --- | --- | --- | --- | --- |
|  |  |  | **A*** | **A*** | **B*** | **B*** | **C*** | **C*** | **Bw** | **Bw** | **C-group** | **C-group** | **DRB1*** | **DRB1*** | **DQ*** | **DQ*** | **2DS2** | **2DL2** | **2DL3** | **2DP1** | **2DL1** | **3DL1** | **2DS4** | **3DS1** | **2DS1** | **2DL5** | **2DS3** | **2DS5** |  |
| Patient | | | 02:01 | 31:01 | 37:01 | 48:01 | 06:02 | 08:01 | 4 | 6 | 2 | 1 | 11:01 | 15:01 | 03:01 | 05:03 | + | + | + | + | + | + | +(F) | - | - | - | - | - | **-** |
| Donor 1 | | | 02:01 | 31:01 | 37:01 | 15:11 | 07:06 | 08:01 | 4 | 4 | 1 | 1 | 11:01 | 15:01 | 03:01 | 05:03 | + | - | - | + | + | + | +(D) | - | + | +(A,B) | + | - | **no** |
| Donor 2 | | | 02:01 | 31:01 | 49:01 | 48:01 | 06:02 | 03:03 | 4 | 6 | 2 | 1 | 11:01 | 15:01 | 03:01 | 05:03 | + | - | + | + | - | + | +(D) | + | - | +(A,B) | - | + | **no** |
|  |  |  | | | | | | | | | | | | | | | | | | | | | | | | | | | |
