# Supplementary material for: Case Report: Cord blood-derived natural killer cells as new potential immunotherapy drug for solid tumor: a case study for endometrial cancer
Source: Front Immunol. 2023 Jun 30;14:1213161. doi: 10.3389/fimmu.2023.1213161 (PMC10348479; doi:10.3389/fimmu.2023.1213161)
Supplement: Supplementary file 3 [file Table_3.docx]

Supplementary TABLE 3. Immunophenotyping of lymphocyte subsets.

| Immune cell type | Surface markers | Normal range | V1 | V2 | V3 | V4 | V5 |
| --- | --- | --- | --- | --- | --- | --- | --- |
| HSC (%) | CD34+ | 0.03-0.70 | 0.01↓ | 0.003↓ | 0.01↓ | 0.01↓ | 0.01↓ |
| HSC (cells/μL) |  | 0.320-3.50 | 2.1 | 0.1359↓ | 0.56 | 0.58 | 0.54 |
| Lymphocytes (%) | CD14-SSLow | 20.0-40.0 | 6.73↓ | 41.46↑ | 32.22 | 36.34 | 31.85 |
| Lymphocytes (cells/μL) |  | 1488-4483 | 1413.3↓ | 1878.13 | 1804.32 | 2107.72 | 1719.9 |
| Monocytes (%) | CD14+ | 3.0-8.0 | 4.24 | 1.69↓ | 5.72 | 7.63 | 9.1↑ |
| Monocytes (cells/μL) |  | 300-800 | 890.4↑ | 76.56↓ | 320.32 | 442.54 | 491.4 |
| Granulocytes (%) | CD14-SShigh | 50.0-70.0 | 88.51↑ | 56.48 | 61.41 | 54.82 | 58.4 |
| T (%) | CD3+ | 50.0-84.0 | 66.67 | 76.16 | 76.04 | 70.8 | 70.08 |
| T (cells/μL) |  | 955-2860 | 942.25↓ | 1430.39 | 1372.01 | 1492.27 | 1205.31 |
| B (%) | CD19+ | 5.0-18.0 | 6.89 | 2.88↓ | 3.03↓ | 4.21↓ | 4.25↓ |
| B (cells/μL) |  | 90-560 | 97.38 | 54.09↓ | 54.67↓ | 88.74↓ | 73.09↓ |
| NK cells (%) | CD56+CD3- | 7.0-40.0 | 17.54 | 13.27 | 12.9 | 17.61 | 18.07 |
| NK cells (cells/μL) |  | 150-1100 | 247.89 | 249.23 | 232.76 | 371.17 | 310.79 |
| NK-T (%) | CD56+CD3+ | 3.0-8.0 | 6.43 | 4.86 | 6.96 | 6.6 | 4.58 |
| γδ T (%) | TCRγδ+CD3+ | 1.0-10.0 | 2.24 | 2.05 | 1.66 | 1.81 | 1.96 |
| T CD4+ (%) | CD3+CD4+ | 27.0-51.0 | 27.47 | 40.19 | 30.37 | 28.70 | 33.92 |
| T CD4+ (cells/μL) |  | 414-1123 | 388.3↓ | 754.82 | 547.98 | 604.96 | 583.37 |
| T CD8+ (%) | CD3+CD8+ | 15.0-44.0 | 32.49 | 30.58 | 40.39 | 36.84 | 32.07 |
| T CD8+ (cells/μL) |  | 238-874 | 459.25 | 574.30 | 728.80 | 776.43 | 551.54 |
| CD4 : CD8 |  | 0.71-2.78 | 0.85 | 1.31 | 0.75 | 0.78 | 1.06 |
| Treg (%) | CD4+CD25+CD127dim | 2.0-10.0 | 4.78 | 9.96 | 6.43 | 6.59 | 6.79 |
| Early activated T cells (%) | CD3+CD69+ | 0.1-3.5 | 1.79 | 1.47 | 1.61 | 1.39 | 1.46 |
| Middle activated T cells (%) | CD3+CD25+ | 10.0-30.0 | 12.45 | 16.55 | 11.09 | 8.74↓ | 13.33 |
| Late activated T cells (%) | CD3+HLA-DR+ | 15.0-25.0 | 40.44↑ | 40.62↑ | 48.42↑ | 44.66↑ | 40.14↑ |
| Naïve CD4+ T (%) | CD3+CD4+CD27+CD45RA- | 10.0-20.0 | 8.05↓ | 12.83 | 9.28↓ | 6.91↓ | 8.99↓ |
| Naïve CD4+T (cells/μL) |  | 154-485 | 113.82↓ | 241.02 | 167.52 | 145.65↓ | 154.64 |
| Effector memory CD4+T (%) | CD3+CD4+CD27-CD45RO+ | 8.0-25.0 | 22.57 | 29.04↑ | 23.72 | 23.22 | 26.13↑ |
| Effector memory CD4+T (cells/μL) |  | 298-683 | 319.04 | 545.41 | 428.07 | 489.31 | 449.46 |
| Naïve CD8+ T (%) | CD3+CD8+CD27+CD45RA- | 10.0-20.0 | 23.43↑ | 19.80 | 19.61 | 22.26↑ | 19.34 |
| Naïve CD8+ T (cells/μL) |  | 152-395 | 331.11 | 371.90 | 353.84 | 469.17↑ | 332.66 |
| Effector memory CD8+ T (%) | CD3+CD8+CD27-CD45RO+ | 2.0-15.0 | 11.99 | 10.78 | 19.41↑ | 13.73 | 12.09 |
| Effector memory CD8+ T (cells/μL) |  | 85-261 | 169.51 | 202.40 | 350.14↑ | 289.35↑ | 208.04 |
| Myeloid DC (%) | Lineage-CD11c+HLA-DR+ | 0.10-0.50 | 0.04↓ | 0.21 | 0.16 | 0.15 | 0.14 |
| Lymphoid DC (%) | Lineage-CD123+HLA-DR+ | 0.05-0.30 | 0.01↓ | 0.04↓ | 0.04↓ | 0.05 | 0.08 |

↑, above normal range; ↓, below normal range.
