# Supplementary material for: Case Report: Cord blood-derived natural killer cells as new potential immunotherapy drug for solid tumor: a case study for endometrial cancer
Source: Front Immunol. 2023 Jun 30;14:1213161. doi: 10.3389/fimmu.2023.1213161 (PMC10348479; doi:10.3389/fimmu.2023.1213161)
Supplement: Supplementary file 4 [file DataSheet_1.pdf]

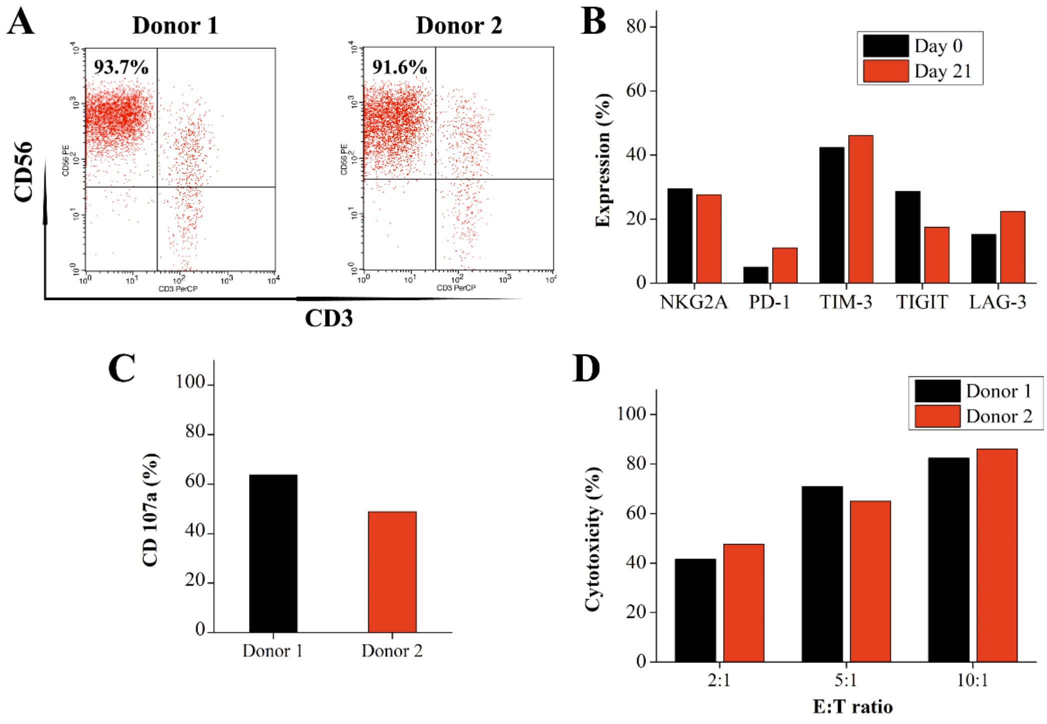

Supplementary data Figure 1 (A) Phenotype of CB-NK cells on day 21 from donor 1 and 2. (B) The expression of inhibitory receptors on days 0 and 21 from donor 1. (C) The expression of CD107a in CB-NK cells on day 21 from donor 1 and 2. (D) Cytotoxicity of CB-NK cells on day 21 from donor 1 and 2.
